# Is Preoperative Patient-Reported Health Status Associated with Mortality after Total Hip Replacement?

**DOI:** 10.3390/ijerph14080899

**Published:** 2017-08-10

**Authors:** Peter Cnudde, Szilard Nemes, Maziar Mohaddes, John Timperley, Göran Garellick, Kristina Burström, Ola Rolfson

**Affiliations:** 1 Swedish Hip Arthroplasty Register, Centre of Registers Västra Götaland, Medicinargatan 18G, SE 413 45 Gothenburg, Sweden; szilard.nemes@registercentrum.se (S.N.); maziar.mohaddes@gmail.com (M.M.); goran.garellick@registercentrum.se (G.G.); ola.rolfson@vgregion.se (O.R.); 2 Department of Orthopaedics, Institute of Clinical Sciences, Sahlgrenska Academy, University of Gothenburg, SE 413 45 Gothenburg, Sweden; 3 Department of Orthopaedics, Hywel Dda University Healthboard, Prince Philip Hospital, Bryngwynmawr, Llanelli SA14 8ED, UK; 4 Hip Unit, Princess Elizabeth Orthopaedic Centre, Royal Devon & Exeter Hospital Barrack Road, Exeter EX2 5DW, UK; jtimperley@mac.com; 5 Department of Learning, Informatics, Management and Ethics (LIME), Health Outcomes and Economic Evaluation Research Group, Karolinska Institutet, Tomtebodavägen 18 a, SE 171 77 Stockholm, Sweden; kristina.burstrom@ki.se; 6 Department of Public Health, Equity and Health Policy Research Group, Karolinska Institutet, Tomtebodavägen 18 a, SE 171 77 Stockholm, Sweden; 7 Health Care Services, Stockholm County Council, Tomtebodavägen 18 a, SE 171 77 Stockholm, Sweden

**Keywords:** total hip replacement, PROMs, EQ-5D, mortality, register

## Abstract

The influence of comorbidities and worse physical status on mortality following total hip replacement (THR) leads to the idea that patient-reported health status may also be a predictor of mortality. The aim of this study was to investigate the relationship between patient-reported health status before THR and the risk of dying up to 5 years post-operatively. For these analyses, we used register data on 42,862 THR patients with primary hip osteoarthritis operated between 2008 and 2012. The relative survival ratio was calculated by dividing the observed survival in the patient group by age- and sex-adjusted expected survival of the general population. Pre-operative responses to the five EQ-5D-3L (EuroQol Group) dimensions along with age, sex, education status, year of surgery, and hospital type were used as independent variables. Results shown that, as a group, THR patients had a better survival than the general population. Broken down by the five EQ-5D-3L dimensions we observed differentiated survival patters. For all dimensions, those reporting extreme problems had higher mortality than those reporting moderate or no problems. In conclusion, worse health status according to the EQ-5-3L before THR is associated with higher mortality up to five years after surgery. EQ-5D-3L responses may be useful in a multifactorial individualized risk assessment before THR.

## 1. Introduction

The success of total hip replacement (THR) for the treatment of symptomatic osteoarthritis (OA) of the hip is well recognised and the operation has been described as the operation of the 20th century [1]. There is an anticipated increased demand in surgical intervention due to the ageing population and the increase in obesity [2,3,4]. The short-term risk of dying following THR is low and has declined over the last decades [5,6,7,8,9]. The 90-day mortality for all patients, including those with surgery due to acute hip fractures and tumours around the hip, has been reported to be 0.7% in Sweden [10]. In England and Wales, the 90-day mortality for patients undergoing hip replacement because of OA was 0.3% in 2011 [5]. Systematic reviews of 30- and 90-day mortality rates in patients undergoing hip or knee replacements found an average 90-day mortality of 0.7% [9,11]. Except for the first post-operative month, elective THR patients have a better relative survival than the general population [12,13,14]. This could be explained by selection bias involved in the decision to proceed with THR; medically fit patients are more likely to be recommended for surgery then those with advanced comorbidities.

The relative mortality risk in the period after surgery is higher in males and younger patients [9,12,14,15,16]. Other factors associated with increased risk of dying up to 90 days post-operatively in THR patients are worse physical status, according to the American Society of Anaesthesiologists (ASA) classification system, and comorbidities such as congestive heart failure, diabetes with complication, renal failure, metastatic cancer, chronic obstructive pulmonary disease, dementia, liver disease, and peptic ulcer disease [5,16].

The influence of comorbidities and physical status on mortality leads to the possibility that patient-reported health status may also be a predictor of mortality after elective THR. The importance of self-reported health status has previously been investigated and been shown to be a predictor of mortality in the elderly population, as well as in the general population over the age of 16 years [17,18]. Although this has not been demonstrated in THR surgery, some studies in other fields like acute coronary syndrome have demonstrated a relationship between worse patient-reported health status and increased mortality [19,20].

The complexity of the interactions between different patient factors and their association with different outcomes (such as pain level, functional status, reoperations, death, and other adverse events) complicates accurate assessments of risks and expected benefits for individual patients. Ideally, modifiable risk factors (such as poor diabetes control, preoperative anaemia, and smoking) should be identified and adjusted in order to minimize risk of complications and improve health outcomes. However, patients should not be excluded or deterred from surgery based on generalised information that may inaccurately overestimate the risks. To facilitate the assessment of risks and benefits, a decision aid providing individualised information on expected outcomes is warranted, which in return could be beneficial in the consenting process. Hip replacement relieves pain and improves mobility, enhancing quality of life for a non-life threatening problem, and patients elect to take the risk involved in surgery. This situation contrasts with life-saving surgery for cancer or cardiac conditions. In the UK, mortality rates for individual surgeons following elective hip replacement surgery have been published, and this had led to much controversy [21,22].

The objective of this study is to investigate the relationship between patient-reported health status as determined by the five EQ-5D-3L dimensions, the self-assessment of overall health using a visual analogue scale (EQ VAS), and hip pain (according to a Visual Analogue Scale (VAS)) before THR and the risk of dying up to five years post-operatively.

Given the low number of events, it is obvious that large datasets are required to investigate if health status, as reported by the patient, is associated with risk of dying after THR. With routine nationwide prospective collection of patient-reported outcome measures (PROMs), the Swedish Hip Arthroplasty Register (SHAR) is well suited to investigate such relationships. The importance of patient-focused registries to improve health, care, and science has been re-iterated in a recent work by Nelson and colleagues and within the field of arthroplasty by Berry [23,24].

## 2. Materials and Methods

### 2.1. Data Sources, Patient Selection, and Measures

**Figure 1:**
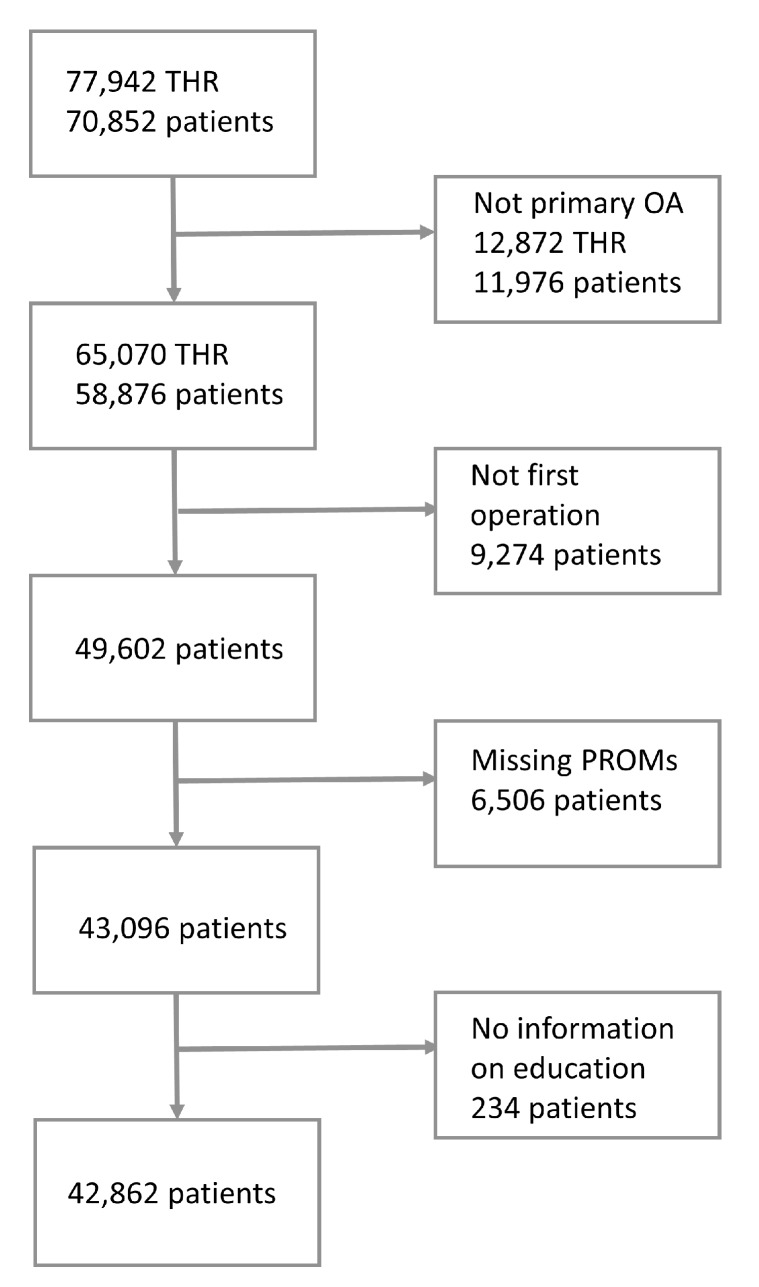
Flowchart study design. THR: total hip replacement; OA: osteoarthritis; PROMs: patient-reported outcome measures.

The SHAR runs a nationwide program collecting PROMs to routinely monitor patients undergoing THR in Sweden [25,26]. PROMs data collection commenced in 2002, was gradually adopted, and reached full active nationwide coverage in 2008. Patients were asked to complete a PROMs survey at their preoperative clinical visit. The response rate of this preoperative survey was 86% [25]. The data available within the SHAR databases has been linked with Statistics Sweden using the Personal Identity Number (PIN) as part of a larger research project and data have subsequently been anonymised. The process of the linkage of the different databases has been described elsewhere [27]. The short PROMs survey includes the EQ-5D-3L instrument (the descriptive system with five health dimensions: mobility, self-care, usual activities, pain/discomfort, and anxiety/depression; and three severity levels: no problems, moderate problems, and severe problems), the EQ VAS (self-assessment of overall health ranging from 0 to 100) and a pain VAS (to assess hip pain ranging from 0 (no pain) to 100 (worst imaginable pain)). These data are prospectively collected prior to surgery as part of the pre-operative visit and recorded in the SHAR database. All patients reported to SHAR and undergoing THR for the first time between 1 January 2008 and 31 December 2012 with a diagnosis of osteoarthritis and complete pre-operative PROMs were included in this study. In order not to violate the assumption of independent observations, it was first decided that a patient could only contribute with data from one primary hip replacement. Subsequently, however, we decided to only include the first hip to reduce heterogeneity. Additional to the PROMs, we obtained data on sex, age at operation, hospital type (university, county, rural or private), date of operation, method of fixation, and date of death, if applicable. The mortality data is obtained by cross-matching with the Swedish Population Register, governed by the Swedish Tax Office [28]. Based on the unique personal identity number, we obtained data on the educational level (highest completed level of education at time of operation) through linkage to Statistics Sweden. We used low, middle, and high levels of education to respectively describe the first 9 years of education (secondary school level), an extra 2 or 3 years (college level), and higher education (university or similar). Using these selection criteria, we identified 42,862 patients for the study group (Figure 1). To a certain extent, the PROMs act as proxies for patient comorbidities. We tested the association between the dimension of the EQ-5D-3L questionnaire and the ASA class (American Society of Anaesthesiologists physical status classification) and the International Classification of Diseases 10th revision (ICD-10) based Elixhauser comorbidity index [29]. 

### 2.2. Statistical Analyses and Software

Group comparison between survivors and deceased patients were conducted with Student’s *t*-test for continuous variables and the χ^2^ test for categorical variables. 

Survival data was summarised and illustrated with the help of relative survival curves. We opted for relative survival over the classical Kaplan-Meier survival curves and Cox Proportional Hazards in order to gain better insight, not only into the differences between the levels of the five EQ-5D-3L dimensions, but also to be able to relate the survival of the patients to the general population.

The relative survival ratio *r*(*t*) is defined as the observed survival in the patient group divided by the expected survival of a comparable group from the general population:

(1) $$r(t)=\frac{S_{O}(t)}{S_{p}(t)}$$

where *S_O_*(*t*) denotes the observed survival in the studied group and *S_P_*(*t*) is the population or expected survival [30]. The population or expected survival was estimated from publicly available mortality tables tabulated for sex and age [31]. The population or expected survival was estimated from publicly available mortality tables maintained by the Human Life-Table Database (http://www.lifetable.de/) and Human Mortality Database (http://www.mortality.org).

Multivariable modelling proceeded with time-transformed Cox Proportional Hazards [32]. EQ VAS and pain VAS were measured in units of 10. Model assumptions were checked with Brownian bridges [33]. We assumed that age might have a non-linear effect on survival. We fitted a model where age was modelled with cubic splines. However, information theoretic assessment with the Bayesian Information Criterion strongly favoured the linear model; thus, this approach was dropped.

We used R v3.4 (R Core team, Vienna, Austria, 2016) for statistical calculations. 

### 2.3. Research Ethics

This study is part of a larger research project with the overall aim of performing a multidimensional outcomes assessment following hip replacement surgery, and developing an instrument to facilitate shared decision-making before hip replacement. All the investigations were carried out following the rules of the Declaration of Helsinki. Ethical review approval was obtained from the Regional Ethical Review Board in Gothenburg, Sweden (entry number 271-14) on 9 April 2014.

In accordance with the Swedish Patient Data Act (2008:355), patients receive information about being registered and have the full right to opt-out. All patients undergoing hip replacement surgery in Sweden are, unless they opt out, per definition enrolled in the scientific studies performed by and at the SHAR.

### 2.4. Availability of Data and Materials

The datasets generated and analysed for the current study are not publicly available but are stored in Secure-online Data Access (SODA) servers within the Registercentrum Vastra Götaland, Gothenburg, Sweden and can be made available upon reasonable request [27].

## 3. Results

Of the 42,862 patients in the cohort, 1346 died in the follow-up period (range = 5.0 years, mean = 2.4 years, Standard Deviation (SD) = 1.4 years). There were statistically significant differences between survivors and deceased regarding sex, age at day of operation, hospital type, the five EQ-5D dimensions, the EQ VAS, the pain VAS, and educational level (Table 1).

**Figure 2:**
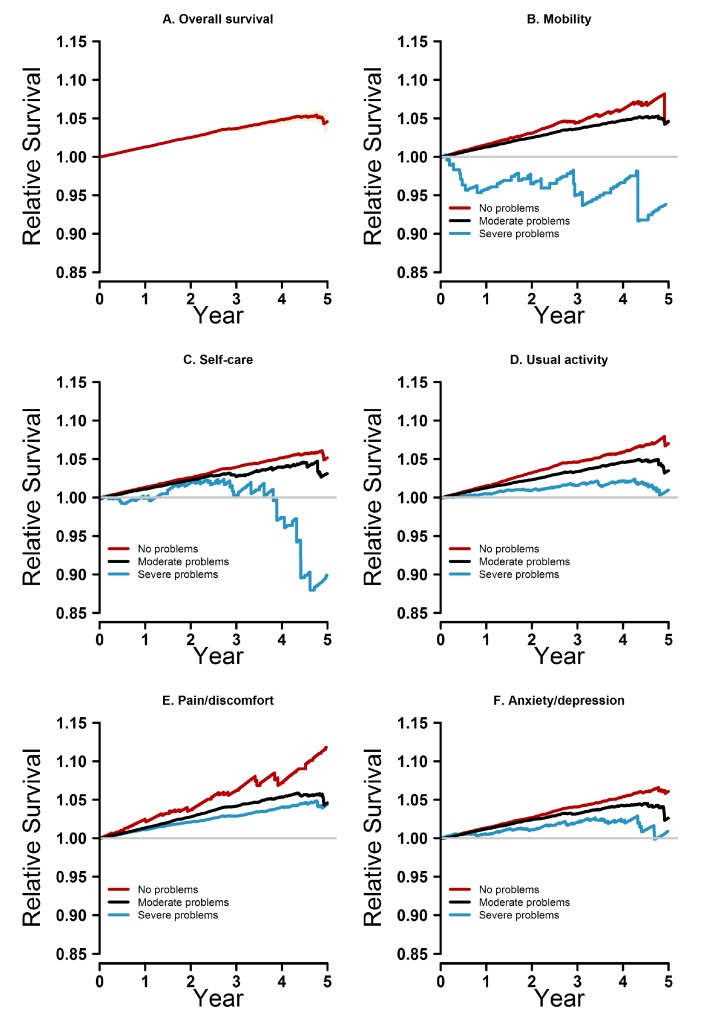
Relative survival curves for the overall study population (**A**) and broken down by the five dimensions of the EQ-5D-3L (**B**–**F**). All our survival analyses are based on recorded PROMs preoperatively.

As a group, THR patients had a better survival rate than the general population (Figure 2).

Females had a better survival rate than males. Of interest, the so-called protective effect of hip replacement on mortality is more profound in the more advanced age group. Compared to patients operated at university hospitals, patients operated at any other type of hospitals had a better survival rate.

Broken down by the five EQ-5D-3L dimensions, we observed differentiated survival patterns (Figure 2).

Patients who reported no problems on any of the EQ-5D-3L dimensions had a better survival rate than the general population and patients who reported moderate or severe problems. Patients who reported moderate problems on any of the EQ-5D-3L dimensions had a better survival rate than the general population and patients who reported severe problems. The worse the patient scored on any of the EQ-5D-3L dimensions, the higher the hazard rates of increased mortality became. Only a relatively small number of patients (*n* = 131) reported severe problems on the mobility dimension, but they were found to have worse survival than the general population; patients who reported severe problems on the self-care dimension had a slight drop in survival probability straight after the operation. In the time span of one to four years after the operation, these patients had a better survival rate than the general population; after year four, the survival chances worsened. Patients who reported severe problems on the dimensions of pain/discomfort, usual activities, and anxiety/depression had a better survival rate than the general population. This pattern was reinforced by the multivariable regression analysis (Table 2).

Reviewing the EQ VAS data, we also discovered an association with lower patient-reported overall health in the non-survival group. The pain VAS was neither clinically nor statistically significantly associated with a difference in survival.

Although we identified an association between a higher obtained educational level and improved survival chances after THR, these differences were not statistically significant and should be interpreted with caution.

**Figure 3:**
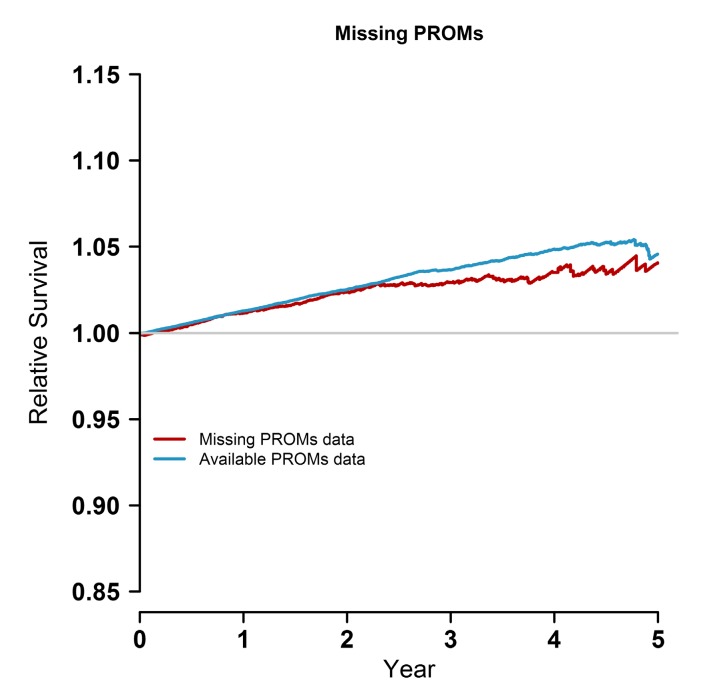
Relative survival curves for the population with missing PROMs and available PROMs. All our survival analyses are based on (present or absent) preoperative recorded PROMs. Although there is a difference between the groups, the difference is not statistically significant (*p* = 0.07).

We undertook a sensitivity analysis for the missing PROMs data (6506 patients) and we did not observe any significant difference in mortality between the two groups, and the baseline data was statistically significant (however, it lacked a clinically significant difference (Table 3 and Figure 3)).

We observed a strong association between the dimensions of the EQ-5D-3L questionnaire and the ASA score (*p* < 0.0001 for all five dimensions) and the Elixhauser index (*p* < 0.0001 for all 5 dimensions).

## 4. Discussion

Our findings suggest a lower mortality in patients operated with THR compared to the general population. This finding is consistent with previous reports [12,13,14]. The reduced mortality risk is probably due to the selection of healthier individuals that are fit enough to undergo a major elective procedure and also have a preoperative medical assessment and optimization. This effect is more profound in the more advanced age group. This is consistent with previous reports of both the Norwegian and Swedish arthroplasty registers and might well be explained by selection bias [14,15]. The worse survival rate after THR in university hospitals compared to the other non-university hospitals has been a constant in the annual reports of the SHAR (https://shpr.registercentrum.se/shar-in-english) and has been attributed to the difference in patient characteristics of the patients treated at university hospitals.

The analysis of the available data points indicates an increased mortality risk for patients who pre-operatively report more problems in the EQ-5D-3L dimensions of mobility and self-care; however, in our data there are only a limited number of patients reporting severe limitations. The level of problems on the dimensions’ usual activities—pain/discomfort and anxiety/depression—and the EQ VAS score had limited influence on the mortality risk. It is very likely that poorer pre-operative mobility is associated with increased incidence of chronic comorbidities like diabetes and cardiopulmonary disease. This assumption is corroborated by the strong association between the EQ-5D-3L dimensions and the ASA classification and Elixhauser comorbidity index. There is evidence linking an increased sedentary lifestyle with increased risk of hospitalization, cardiovascular disease, and mortality [34]. Jämsen et al. described a significant association between poorer mobility (inability to walk/walking indoors only) and increased risk of early mortality in their single centre-analysis of 1998 hip and knee replacements [16]. Nüesch et al. [35] described the influence of poor mobility on mortality in the presence of osteoarthritis of the hip. They performed a population cohort study in the southwest of England and reported the presence of walking disability as a major risk factor for increased mortality, while pain and depression had a limited influence. In the field of heart failure no association was found between self-reported decreased ability to self-care and increased mortality [36]. To the best of our knowledge, no previous reports have been found on the influence of problems with self-care on mortality in patients undergoing elective THR surgery. There has been some evidence on the influence of problems with anxiety and/or depression on mortality in patients following THR, where most studies report a lower mortality in patients with anxiety and depression [37,38]. In contrast, we found a small negative influence of self-reported problems in the anxiety or depression dimension on survival after elective THR, although this relationship did not reach statistical significance in the multivariable regression analysis. The influence of the pre-operative hip pain VAS score on post-operative mortality is negligible and likely not of any clinical nor statistical significance. Asberg et al. were unable to identify a difference in all-cause mortality between individuals with and without chronic musculoskeletal complaints in their prospective population-based cohort study, as well as their systematic review [39,40].

The findings of our study are in agreement with the historical studies from Sweden and Manitoba, based on a general population, and do suggest there is a relationship between worse self-reported health and an increased risk of dying earlier following hip replacement surgery [17,18]. Bliemel et al. studied the influence of pre-fracture quality of life and early mortality (within one year) and found there was an association between surviving the first postoperative year and the EQ-5D score [41].

There is an increased awareness of the importance and validity of arthroplasty registers and PROMs [42,43,44,45]. Arthroplasty registers traditionally report revision or mortality as the endpoint in measuring success after joint replacement, but it is accepted that revision is only a single and relatively crude marker of the success of the procedure. In a recent study comparing different hip arthroplasty registers it was noted that only few registers have so far been collecting PROMs data, despite the fact that these scores may be highly relevant in measuring the success of the intervention [43]. A change in the type of data recorded in joint registries has been advocated [46,47].

The influence of pre-operative PROMs on postoperative PROMs has been described, but so far, no association between pre-operative PROMs and mortality has been described [48,49]. With an increased interest in outcomes measurement and early postoperative mortality, it is important to realise that many factors, patient-related as well as procedure-related, might influence the outcome [9,16]. The risk of dying is certainly an important consideration for the patient and should be discussed with the individual patient as part of the consenting process. 

The importance of using pre-operative recorded scores within orthopaedics was published by Jansson et al. as he described the possibility of using the EQ-5D instrument in the pre-operative setting within orthopaedic surgery, and specifically found that the postoperative scores following elective THR were improving and reaching levels of an age- and sex-matched population [50]. They suggested that the EQ-5D could be used as part of the discussions with the patients in the pre-operative setting. Using self-rated health and its association with risk of dying is likely to improve the understanding of the risk to life to the patient. Discussions should be centered on the individual situation and risk to an individual patient, and as such the increased mortality risk in patient with poorer pre-operative mobility and increased problems with self-care might well be worth mentioning [51].

A recent decision by the UK Department of Health to publish mortality figures after elective knee- and hip replacements for individual surgeons and hospitals highlights the need to consider pre-operatively-gathered outcome scores in order to explain potential variations and poorer results [21].

We used the information of a nationwide surveillance program, using prospectively collected data to analyse the association between the pre-operatively collected PROMs and the mortality up to five years after THR surgery.

The SHAR has a recognized high coverage and completeness of data (>98%) and a high response rate for the pre-operative PROMs collection (86%). We have also studied the relationship of non-responders of the PROMs program in the postoperative period and are aware that non-responders are usually having worse scores. A difference between preoperative non-responders and responders, and the observed mortality, could not be established.

As with some of the observational and register studies there is an absence of a control group, and as such we could not compare what would have happened to the patients with an increased risk of dying if no surgery would have been performed. The EQ-5D-3L (none, moderate, extreme) has only been recently (2016) upgraded to the EQ-5D-5L (none, minimal, moderate, many, extreme) within the SHAR. We are aware that the five-level survey could well cause less ceiling effect and add more nuance to this study [52].

## 5. Conclusions

It is increasingly recognised that registries are an essential tool to improve healthcare and science. As with all observational studies, it is difficult to prove causation, but we were able to provide associations between pre-operative, patient-reported factors and postoperative mortality. Patients reporting severe problems in mobility, self-care, and usual activity prior to an elective THR for primary osteoarthritis have a higher mortality up to five years after surgery. EQ-5D-3L responses may be useful in a multifactorial, individualized risk assessment before THR. Whilst this study does not need to be seen as a deterrent to performing surgery, it helps identify patients and patient groups that are more at risk of an earlier terminal event, compared to an age- and sex-matched population. Identifying preoperatively patients at increased risk, based on EQ-5D responses, improves the engagement between the patient and the surgeon for the decision-making process and may further improve the outcomes after THR.

## Figures and Tables

**Table 1:** Demographics and pre-operative, health-related quality of life of the 42,862 total hip replacement patients. The data is summarized as absolute numbers and percentages for discrete variables and means, and standard deviations (SDs) for continuous variables.

		Alive	Dead	*p*-Value
		*n* = 41,516	*n* = 1346	
Mobility (%)	No problems	3210 (7.7)	52 (3.9)	<0.001
	Moderate problems	38,190 (92.0)	1279 (95.0)	
	Severe problems	116 (0.3)	15 (1.1)	
Self-care (%)	No problems	32,066 (77.2)	910 (67.6)	<0.001
	Moderate problems	9102 (21.9)	403 (29.9)	
	Severe problems	348 (0.8)	33 (2.5)	
Usual activities (%)	No problems	16,086 (38.7)	460 (34.2)	<0.001
	Moderate problems	21,125 (50.9)	684 (50.8)	
	Severe problems	4305 (10.4)	202 (15.0)	
Pain/discomfort (%)	No problems	631 (1.5)	16 (1.2)	<0.001
	Moderate problems	23,822 (57.4)	706 (52.5)	
	Severe problems	17,063 (41.1)	624 (46.4)	
Anxiety/depression (%)	No problems	23,963 (57.7)	711 (52.8)	<0.001
	Moderate problems	16,079 (38.7)	568 (42.2)	
	Severe problems	1474 (3.6)	67 (5.0)	
EQ VAS score (SD)		54.77 (22.17)	50.61 (21.76)	<0.001
Pain VAS score (SD)		62.39 (15.91)	62.67 (17.34)	0.53
Females (%)		23,358 (56.3)	633 (47.0)	<0.001
Age (SD)		67.70 (10.09)	75.76 (8.83)	<0.001
Educational level (%)	low	14,018 (33.8)	658 (48.9)	<0.001
	middle	17,038 (41.0)	466 (34.6)	
	high	10,460 (25.2)	222 (16.5)	
Hospital (%)	University	3018 (7.3)	117 (8.7)	<0.001
	County	13,026 (31.4)	464 (34.5)	
	Rural	17,490 (42.1)	603 (44.8)	
	Private	7982 (19.2)	162 (12.0)	
Fixation (%)	Cemented	28,237 (68.4)	1168 (87.0)	<0.001
	Uncemented	6363 (15.4)	58 (4.3)	
	Hybrid	536 (1.3)	12 (0.9)	
	Reverse hybrid	5503 (13.3)	102 (7.6)	
	Resurfacing	639 (1.5)	2 (0.1)	

**Table 2:** Results of the relative survival regression analysis on mortality after total hip replacement. The results are presented as Hazard Rates (HR) and associated 95% confidence intervals (CI).

		HR	95% CI
Mobility	No problems	ref	
	Moderate problems	1.46	1.09–1.96
	Severe problems	2.65	1.43–4.92
Self-care	No problems	ref	
	Moderate problems	1.15	1.01–1.31
	Severe problems	1.57	1.08–2.29
Usual activity	No problems	ref	
	Moderate problems	1.05	0.93–1.20
	Severe problems	1.28	1.06–1.56
Pain/discomfort	No problems	ref	
	Moderate problems	1.07	0.64–1.77
	Severe problems	1.20	0.71–2.00
Anxiety/depression	No problems	ref	
	Moderate problems	1.09	0.96–1.22
	Severe problems	1.24	0.95–1.62
EQ VAS (in units of 10)		0.95	0.92–0.98
Pain VAS (in units of 10)		0.96	0.92–1.01
Sex	Male	ref	
	Female	0.86	0.76–0.96
Age		0.96	0.95–0.97
Operation Year		0.91	0.86–0.96
Education	Low	ref	
	Middle	0.93	0.83–1.06
	High	0.85	0.73–1.01
Hospital	University	ref	
	County	0.79	0.65–0.97
	Rural	0.82	0.67–1.00
	Private	0.72	0.56–0.91
Fixation	Cemented	ref	
	Uncemented	0.60	0.45–0.81
	Hybrid	0.80	0.45–1.43
	Reverse hybrid	0.94	0.76–1.16
	Resurfacing	0.20	0.05–0.84

**Table 3:** Demographics, socioeconomics, and surgery-related data of 43,096 total hip replacement patients with PROMs data, and the 6506 total hip replacement patients without PROMs data. The data is summarized as absolute numbers and percentages for discrete variables, and means and standard deviations for continuous variables.

		Missing PROMs	Available PROMs	*p*-Value
		*n* = 6506	*n* = 43,096	
Females (%)		3676 (56.5)	24,139 (56.0)	0.466
Age (SD)		68.59 (11.16)	67.97 (10.16)	<0.001
Educational level (%)	Low	2407 (37.0)	14,676 (34.1)	<0.001
	Middle	2557 (39.3)	17,504 (40.6)	
	High	1453 (22.3)	10,682 (24.8)	
	Missing	89 (1.4)	234 (0.5)	
Hospital (%)	University	687 (10.6)	3154 (7.3)	<0.001
	County	2288 (35.2)	13,571 (31.5)	
	Rural	2239 (34.4)	18,194 (42.2)	
	Private	1292 (19.9)	8177 (19.0)	
Fixation (%)	Cemented	4226 (65.0)	29,594 (68.7)	<0.001
	Uncemented	1099 (16.9)	6451 (15.0)	
	Hybrid	107 (1.6)	551 (1.3)	
	Reverse hybrid	883 (13.6)	5627 (13.1)	
	Resurfacing	83 (1.3)	643 (1.5)	
